# A humanized antibody for imaging immune checkpoint ligand PD-L1 expression in tumors

**DOI:** 10.18632/oncotarget.7143

**Published:** 2016-02-01

**Authors:** Samit Chatterjee, Wojciech G. Lesniak, Matthew Gabrielson, Ala Lisok, Bryan Wharram, Polina Sysa-Shah, Babak Behnam Azad, Martin G. Pomper, Sridhar Nimmagadda

**Affiliations:** ^1^ Russell H. Morgan Department of Radiology and Radiological Science, Johns Hopkins University, Baltimore, MD, USA; ^2^ Sidney Kimmel Comprehensive Cancer Center, Johns Hopkins University, Baltimore, MD, USA

**Keywords:** MPDL3280A, immunotherapy, immune escape, molecular imaging, personalized medicine

## Abstract

Antibodies targeting the PD-1/PD-L1 immune checkpoint lead to tumor regression and improved survival in several cancers. PD-L1 expression in tumors may be predictive of response to checkpoint blockade therapy. Because tissue samples might not always be available to guide therapy, we developed and evaluated a humanized antibody for non-invasive imaging of PD-L1 expression in tumors. Radiolabeled [^111^In]PD-L1-mAb and near-infrared dye conjugated NIR-PD-L1-mAb imaging agents were developed using the mouse and human cross-reactive PD-L1 antibody MPDL3280A. We tested specificity of [^111^In]PD-L1-mAb and NIR-PD-L1-mAb in cell lines and in tumors with varying levels of PD-L1 expression. We performed SPECT/CT imaging, biodistribution and blocking studies in NSG mice bearing tumors with constitutive PD-L1 expression (CHO-PDL1) and in controls (CHO). Results were confirmed in triple negative breast cancer (TNBC) (MDAMB231 and SUM149) and non-small cell lung cancer (NSCLC) (H2444 and H1155) xenografts with varying levels of PD-L1 expression. There was specific binding of [^111^In]PD-L1-mAb and NIR-PD-L1-mAb to tumor cells *in vitro*, correlating with PD-L1 expression levels. In mice bearing subcutaneous and orthotopic tumors, there was specific and persistent high accumulation of signal intensity in PD-L1 positive tumors (CHO-PDL1, MDAMB231, H2444) but not in controls. These results demonstrate that [^111^In]PD-L1-mAb and NIR-PD-L1-mAb can detect graded levels of PD-L1 expression in human tumor xenografts *in vivo*. As a humanized antibody, these findings suggest clinical translation of radiolabeled versions of MPDL3280A for imaging. Specificity of NIR-PD-L1-mAb indicates the potential for optical imaging of PD-L1 expression in tumors in relevant pre-clinical as well as clinical settings.

## INTRODUCTION

Tumor cells avoid the immune response by exploiting immune checkpoints through expression of immunosuppressive molecules, recruitment of suppressive immune cell populations and secretion of soluble suppressive factors [1]. Immune checkpoints are inhibitory pathways integral to the immune system, which are critical for modulating the immune response to maintain self-tolerance and prevent autoimmunity. Most immune checkpoints involve ligand-receptor interactions, the inhibition of which repeal the immunosuppression exerted by tumor cells, leading to recognition and destruction of tumor cells by the immune system [1, 2]. Treatment with antibodies blocking immune checkpoint pathway ligands and receptors has shown durable tumor regression and improved patient survival [1, 3-6]. Programmed death ligand-1 (PD-L1, B7-H1 or CD274) is emerging as a central player in immune checkpoint therapies.

PD-L1, a 290 amino acid type I transmembrane glycoprotein, is the primary ligand of programmed death-1 (PD-1). Binding of PD-L1 to PD-1 suppresses T-cell immune activity and restricts tumor cell killing [1, 7]. PD-L1 expression is upregulated in the tumor microenvironment (TME), (possibly as an immune-evasion mechanism) [8] and may be due to: (i) increased PD-L1 expression on tumor cells by intrinsic oncogenic events (e.g., loss of phosphatase and tensin homolog) [9], (ii) tumor cell PD-L1 induction in response to T-cell secreted interferon-gamma [10] and (iii) PD-L1 expression on accumulated myeloid cells and/or dendritic cells that have suppressive effects on T-cells [11]. In cancer patients, there is a strong correlation between PD-L1 expression on tumor cells and poor prognosis [7]. Furthermore, in PD-1 and PD-L1 targeted therapies across multiple cancer types, there is a strong positive correlation between pre-treatment PD-L1 expression in TME and therapeutic response to PD-1/PD-L1 pathway inhibitions [6, 12, 13].

Antibodies targeting PD-L1 (MPDL3280A, MEDI4736 and BMS-936559) have demonstrated anti-tumor activity in diverse tumor types, including renal cell carcinoma (RCC) [14], advanced melanoma [6], non-small cell lung cancer (NSCLC) [6, 14, 15], and bladder cancer [13] among others [2, 16]. Nearly 45% of patients with PD-L1 positive TME show an objective response (OR) following immune checkpoint blockade. Those observations suggest that expression of PD-L1 in tissue biopsies is a valuable biomarker for immune checkpoint therapies [2, 16, 17], but tissue samples are often impractical to obtain, particularly in the setting of recurrent and metastatic disease. Thus, non-invasive detection of the changes in PD-L1 expression in the TME may guide patient management.

MPDL3280A (Atezolizumab) is a humanized monoclonal IgG1k antibody with high affinity for both human and mouse PD-L1, with dissociation constants (*K*_d_) of 0.43 nM and 0.13 nM, respectively [13, 18]. In clinical trials, MPDL3280A proved effective in several tumor types including NSCLC, RCC and TNBC [6, 13].

The clinical efficacy of MPDL3280A prompted us to investigate its application for non-invasive detection of PD-L1 expression in tumors. We developed radiolabeled and near-infrared (NIR) dye-tagged analogs of MPDL3280A (PD-L1-mAb) and tested their specificity in Chinese hamster ovary tumors with constitutive PD-L1 expression. Observations from those studies were then validated in orthotopic and subcutaneous TNBC and NSCLC human tumor xenografts with varying levels of expression of PD-L1. Specific accumulation of SPECT and optical imaging signal intensity was seen in tumors with high PD-L1 expression. Specificity of the uptake was confirmed by *ex vivo* biodistribution and immunohistochemistry studies. Our results with a humanized PD-L1-mAb confirm PD-L1-specific signal accumulation in multiple tumor models. These studies demonstrate imaging of PD-L1 *in vivo* and provide the basis for clinical translation of derivatives of anti-PD-L1 antibodies for imaging.

## RESULTS

### Radiolabeling

Radiolabeled [^111^In]PD-L1-mAb was produced with a specific activity of 4.8±0.65 μCi/μg with > 98% and ∼75% radiochemical purity and immunoreactive fraction, respectively (Supplementary Figure 1A-1C).

### PD-L1 antibody imaging probes show specificity *in vitro*

**Figure 1:**
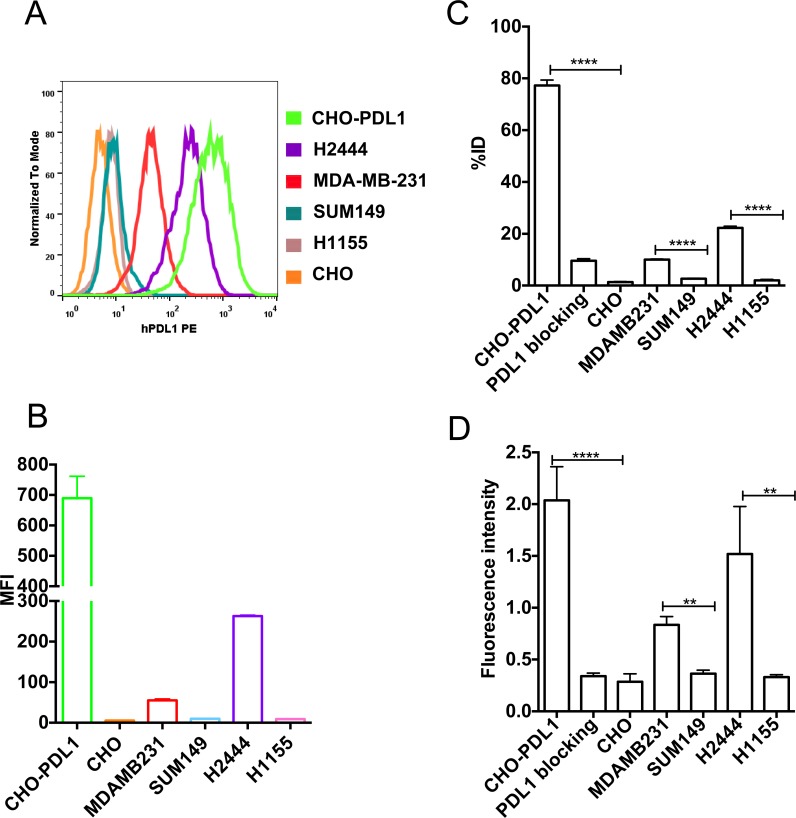
*In vitro* specificity of [^111^In]PD-L1-mAb and NIR-PD-L1-mAb Flow cytometry analysis of various cell lines for cell surface PD-L1 expression **A.** Representative mean fluorescence intensity (MFI) values for PE conjugated anti-human PD-L1 antibody binding to various cell lines **B.** *In vitro* uptake of [^111^In]PD-L1-mAb in CHO-PDL1, CHO, MDAMB231, SUM149, H2444 and H1155 cells incubated with 37 kBq (1 μCi)/100 μL of [^111^In]PD-L1-mAb at 37°C for 1h **C.** *In vitro* uptake of NIR-PD-L1-mAb in CHO-PDL1, CHO, MDAMB231, SUM149, H2444 and H1155 cells incubated with 1 μM NIR-PD-L1-mAb at 37°C for 1 h **D.** Data are represented as percentage of incubated dose (%ID) per million cells and represent mean values of three experiments ± SEM. The significance of the value is indicated by asterisks (*) and the comparative reference is the control or low PD-L1 expression cell line. **p < 0.01, ****p < 0.0001.

We selected CHO-PDL1, CHO, MDAMB231, SUM149, H2444, and H1155 cells to analyze the specificity of PD-L1-mAb to detect graded levels of PD-L1 expression. The percentage of cells that were positive for cell surface PD-L1 expression varied markedly, as shown by flow cytometry: CHO-PDL1 (99%), H2444 (95%), MDAMB231 (27%), SUM149 (0.1%), H1155 (0%) and CHO cells (0%). Furthermore, PD-L1 expression levels, as shown by mean fluorescence intensity (MFI), also varied between cell types. The graded expression profile was: CHO-PDL1 > H2444 > MDAMB231 > SUM149 > H1155 > CHO (Figure 1A and 1B, Supplementary Figure 2). The uptake values, determined by incubation of [^111^In]PD-L1-mAb with 10^6^ cells of each cell line, showed: CHO-PDL1 > H2444 > MDAMB231 > SUM149 > H1155 > CHO (Figure 1C).

Blocking of [^111^In]PD-L1-mAb binding to CHO-PDL1 cells, by addition of 10-fold molar equivalent excess of unlabeled antibody, reduced radioactivity uptake by 75%, indicating that [^111^In]PD-L1 mAb binding is specific. A similar uptake profile was observed with NIR-PD-L1-mAb (Figure 1D). These *in vitro* data indicate that PD-L1 targeted antibody-based imaging probes can be used to detect graded levels of PD-L1 expression in cancer cells.

### PD-L1 mAb shows specific uptake in tumors with stable PD-L1 expression

**Figure 2:**
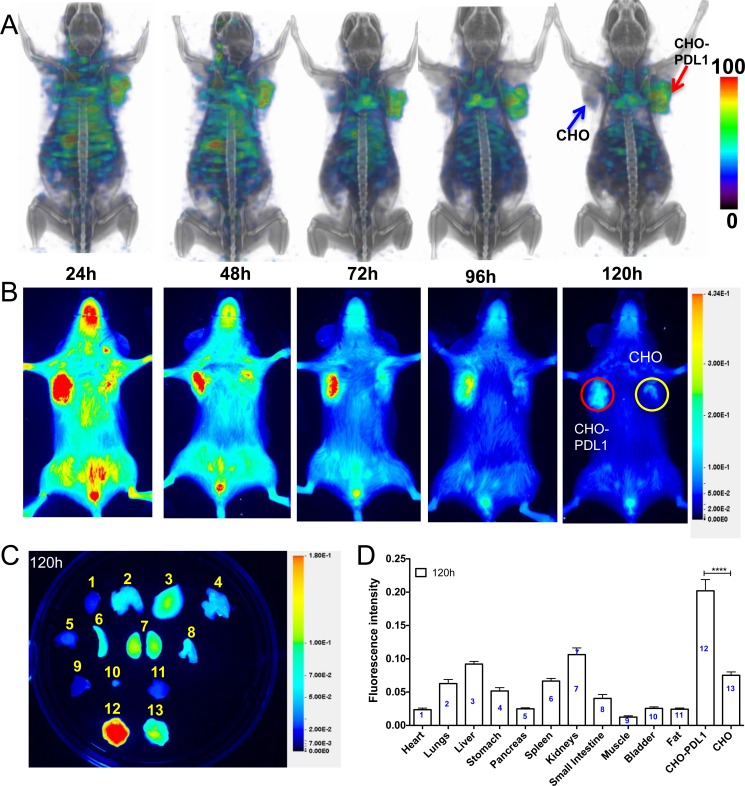
Imaging PD-L1 expression in subcutaneous CHO xenografts with [^111^In]PD-L1-mAb and NIR-PD-L1-mAb NSG mice with CHO and CHO-PDL1 xenografts were administered intravenously with 14.8 MBq (400 μCi) of [^111^In]PD-L1-mAb or 22 μg of NIR-PD-L1-mAb and images were acquired at 24, 48, 72, 96 and 120 h after the injection of the mAbs. 3D volume rendered whole body SPECT/CT images demonstrate specific accumulation of activity in the CHO-PDL1 tumors **A.** Optical images acquired in the 800nm NIR channel **B.** *Ex vivo* biodistribution analysis representative image **C.** and semi-quantitative analysis of fluorescence intensity at 120 h after the injection of NIR-PD-L1-mAb (*n* = 5) **D.** Column numbers in panel D represent the tissue numbers in panel C. All the SPECT images were decay corrected and adjusted to the same maximum value to show the clearance of the imaging agent. The significance of the value is indicated by asterisk (*) and the comparative reference is the tumor with low PD-L1 expression. Arrows and circles depict tumors. ****p < 0.0001.

Several factors, as discussed in the introduction, contribute to changes in PD-L1 expression in the tumor microenvironment. Accordingly, we first established the *in vivo* specificity of [^111^In]PD-L1-mAb in tumors with constitutive PD-L1 expression. SPECT/CT images acquired over 120 h demonstrated substantial and specific accumulation of [^111^In]PD-L1-mAb in CHO-PDL1 tumors but not in control CHO tumors (Figure 2A). Radioactivity accumulation could also be seen in the lungs, liver, and spleen.

To validate the imaging results and to establish protein dose requirements, a protein-dose escalation biodistribution study was performed. In mice injected with [^111^In]PD-L1-mAb alone, at 48 h, the highest uptake (in %ID/g) was in spleen (23.5±8.2), followed by CHO-PDL1 tumor (4.7±0.7) and liver (8.2±4.5) (Table 1). In contrast, in mice co-injected with 10, 30 and 90 μg of unlabeled antibody, the highest uptake was in CHO-PDL1 tumors, in which %ID/g was 13±4 12.5±0.6, and 13.3±1.6 for 10, 30 and 90 μg dose cohorts, respectively. In the spleen, uptake significantly decreased with increased antibody dose, suggesting that spleen acts as a sink for this specific antibody. There were no significant changes in other tissues, nor in tumor or tissue uptake between 30 and 90 μg dose cohorts. Based on the high CHO-PDL1 tumor-to-muscle (21.7±1.3) and CHO-PDL1 tumor-to-blood ratios (2.5±0.1), all other biodistribution studies were performed with 30 μg co-injection of unlabeled antibody.

**Table 1:** Biodistribution of ^111^In-PD-L1-mAb in NSG mice with CHO-PDL1 and control CHO tumors

Tissue	^111^In-PD-L1-mAb [40 μCi/8.3 μg]					
	48h pi					120h pi
	---	10 μg^a^	30 μg^a^	90 μg^a^	1.5 mg^a^	30 μg^a^
**Blood**	0.90 ±0.13	4.46 ±0.65	4.99 ±0.59	6.95 ±0.79	5.75 ±0.1	2.93 ±0.42
**Heart**	2.34 ±1.76	1.31 ±0.91	1.64 ±0.07	2.22 ±0.59	1.92 ±0.1	1.31 ±0.18
**Lungs**	5.95 ±0.72	3.55 ±0.63	3.12 ±0.29	3.99 ±1.07	3.23 ±0.01	4.09 ±0.48
**Liver**	8.24 ±4.52	5.03 ±0.13	3.62 ±0.51	3.5 ±0.40	4.66 ±0.21	5.07 ±0.4
**Stomach**	2.86 ±0.71	1.09 ±0.20	1.13 ±0.18	1.12 ±0.19	0.99 ±0.08	1.44 ±0.11
**Pancreas**	1.96 ±0.41	0.73 ±0.11	0.72 ±0.09	0.72 ±0.12	0.72 ±0.05	1.12 ±0.34
**Spleen**	23.55 ±8.2	7.44 ±2.78	4.75 ±0.38	4.02 ±0.63	21.2 ±6.05	18.23 ±4.85
**Kidney**	12.91 ±6.51	2.64 ±0.19	2.28 ±0.26	2.39 ±0.31	7.55 ±9.32	4.09 ±1.58
**Small Int.**	11.81 ±1.55	1.87 ±0.3	1.22 ±0.25	1.02 ±0.20	0.87 ±0.01	2.88 ±0.19
**Muscle**	0.61 ±0.06	0.58 ±0.11	0.58 ±0.06	0.51 ±0.01	0.61 ±0.06	0.43 ±0.07
**Bladder**	2.08 ±0.37	2.28 ±0.41	1.71 ±1.25	2.75 ±0.42	2.67 ±0.13	2.18 ±0.18
**Fat**	1.33 ±0.53	2.61 ±1.53	0.62 ±0.09	0.65 ±0.28	0.75 ±0.18	0.86 ±0.40
**CHO-PDL1**	4.68 ±0.69	14.36 ±3.75^b^	12.51 ±0.99^b^	13.93 ±1.01^b^	5.91 ±0.35^c^	16.47 ±3.01^b^
**CHO**	3.44 ±0.59	3.18 ±0.29	2.68 ±0.27	2.87 ±1.11	3.05 ±1.03	2.36 ±0.59
**CHO-PDL1:** **muscle**	5.37 ±0.95	24.62 ±8.42	21.71 ±1.28	24.25 ±3.18	9.75 ±0.98	38.22 ±3.56
**CHO-PDL1:** **blood**	5.22 ±0.27	3.36 ±0.94	2.51 ±0.11	2.12 ±0.52	1.03 ±0.05	5.79 ±1.87
**CHO:** **Muscle**	3.95 ±0.78	4.98 ±1.61	4.66 ±0.66	4.77 ±0.77	5.03 ±1.65	5.45 ±0.86
**CHO:** **blood**	3.83 ±0.25	0.67 ±0.15	0.11 ±0.11	0.41 ±0.06	0.53 ±0.18	0.83 ±0.32

pi - post injection;

a amount of unmodified mAb co-injected with radiotracer

b *p* < 0.0001, comparative reference is CHO tumor

c *p<* 0.001, comparative reference is CHO-PDL1 tumor uptake with 30 μg parent antibody dose.

We further evaluated the temporal changes in [^111^In]PD-L1-mAb (30 μg dose) biodistribution in the CHO-PDL1 tumor model at 120 h (Table 1). There was a substantial accumulation of radioactivity in CHO-PDL1 tumors (16.5±3.0 %ID/g). Blood pool radioactivity was reduced and spleen uptake was increased at 120h. In all other tissues, there was no significant difference in %ID/g between 48 and 120 h time points. Thus for CHO-PDL1 tumors, by 120 h, there was high tumor-to-muscle (38.7±8.0) and tumor-to-blood ratios (5.8±1.9), accounting for the tumor-specific high image contrast seen in the SPECT/CT images.

*In vivo* PD-L1 specificity of the antibody was further validated in mice that received 1.5 mg of unlabeled PD-L1 mAb as blocking dose, in which there was a 65% decrease (*P <* 0.001) in CHO-PDL1 tumor radioactivity uptake (Table 1). That tumor specific radioactivity uptake was corroborated by intense immunoreactivity observed in CHO-PDL1 tumors (Supplementary Figure 3).

Similarly, in NIR-PD-L1-mAb-injected mice, signal intensity was consistent and substantial in CHO-PDL1 tumors during the 120 h study (Figure 2B). Biodistribution studies at 120 h showed a 3-fold increase in fluorescence signal intensity in the CHO-PDL1 tumors compared to CHO control tumors (*P <* 0.0001, Figure 2C and 2D). Liver, lungs, and kidneys also showed a distinct accumulation of fluorescence signal. These results show that NIR-PD-L1-mAb can be used to specifically detect tumor PD-L1 expression *in vivo*.

These data demonstrate: (i) [^111^In]PD-L1-mAb and NIR-PD-L1-mAb detect endogenous tumor PD-L1 expression, and (ii) *in vivo* specificity and dose dependent distribution of the anti-PD-L1 antibody.

### Increased uptake of PD-L1 mAb in TNBC xenografts with high PD-L1 expression

**Figure 3:**
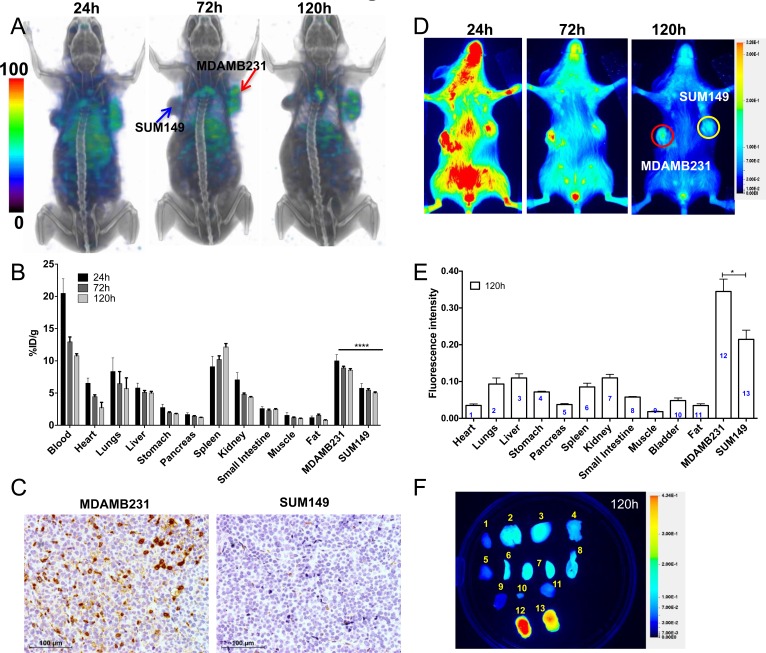
Imaging PD-L1 expression in orthotopic breast cancer xenografts with [^111^In]PD-L1-mAb and NIR-PD-L1-mAb NSG mice with orthotopic MDAMB231 and SUM149 xenografts were administered intravenously with 14.8 MBq (400 μCi) of [^111^In]PD-L1-mAb or 22 μg of NIR-PD-L1-mAb and images were acquired at 24, 72, and 120 h after the injection of the mAbs. 3D volume rendered whole body SPECT/CT images demonstrating specific accumulation of activity in the MDAMB231 tumors **A.** *Ex vivo* biodistribution analysis of the [^111^In]PD-L1-mAb at 24 h, 72 h and 120 h after injection, in the same tumor models **B.**. Immunohistochemical analysis for PD-L1 expression demonstrating intense immunoreactivity in MDAMB231 tumors compared to SUM149 tumors **C.** Optical images acquired in the 800 nm NIR channel show specific accumulation of fluorescence signal in the MDAMB231 tumors **D.** *Ex vivo* biodistribution analysis of fluorescence intensity in tissues (*n* = 4) **E.** and representative image at 120 h after the injection of NIR-PD-L1-mAb **F.** Column numbers in panel E represent the tissue numbers in panel **F**. SPECT images were decay corrected and adjusted to the same maximum value to show the clearance of the imaging agent.. The significance of the value is indicated by asterisk (*) and the comparative reference is the tumor with low PD-L1 expression. Arrows and circles depict tumors. *p < 0.05, ****p < 0.0001.

SPECT/CT images of mice with MDAMB231 and SUM149 TNBC xenografts that were injected with [^111^In]PD-L1-mAb showed high radioactivity accumulation in the MDAMB231 tumors compared to SUM149 (Figure 3A). The distribution profile in other tissues was similar to that of the CHO tumor model. Biodistribution studies indicated highest radioactivity in MDAMB231, blood and spleen (Figure 3B). The %ID/g values for the MDAMB231 and SUM149 tumors were 8.9±0.26 and 5.5±0.21, respectively at 72 h after [^111^In]PD-L1-mAb injection. The tumor-to-muscle and tumor-to-blood ratios for MDAMB231 tumors at 120 h were 8.2±0.8, and 0.79±0.06, respectively. IHC evaluation showed strong PD-L1 immunoreactivity in MDAMB231 but not in SUM149 tumors (Figure 3C), validating [^111^In]PD-L1-mAb specificity.

Optical imaging of NIR-PD-L1-mAb distribution in the MDAMB231 and SUM149 xenografts during 120 h showed consistent and high fluorescence intensity in the MDAMB231 tumors compared to the SUM149 tumors (Figure 3D). *Ex vivo* analysis of the same mice confirmed the imaging observations (Figure 3E). In addition to the MDAMB231 tumors, increased fluorescence intensity was observed in SUM149 tumors, liver and lungs (Figure 3E and 3F). These studies establish that [^111^In]PD-L1-mAb and NIR-PD-L1-mAb have the specificity to detect endogenous PD-L1 expression in TNBC tumors.

### Increased uptake of PD-L1 mAb in NSCLC xenografts with high PD-L1 expression

**Figure 4:**
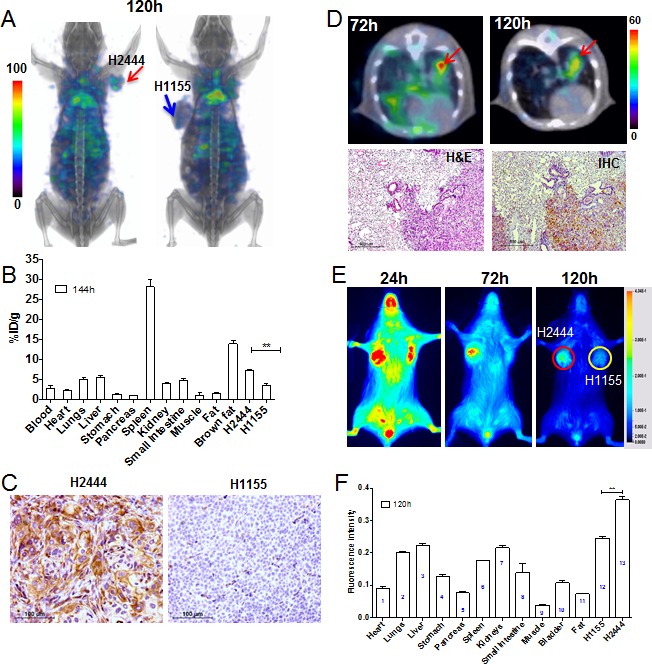
Imaging PD-L1 expression in subcutaneous and orthotopic lung cancer xenografts with [^111^In]PD-L1-mAb and NIR-PD-L1-mAb NSG mice with subcutaneous H2444 or H1155 xenograft were administered intravenously with 14.8 MBq (400 μCi) of [^111^In]PD-L1-mAb or 22 μg of NIR-PD-L1-mAb and images were acquired at the specified time after the injection of the mAbs. 3D volume rendered whole body images demonstrating specific accumulation of activity in the H2444 tumors at 120 h and not in the H1155 tumors **A.** *Ex vivo* biodistribution analysis of the [^111^In]PD-L1-mAb, at 144 h after injection, in the same tumor models **B.** Immunohistochemical analysis for PD-L1 expression demonstrating intense immunoreactivity in H2444 tumors compared to H1155 tumors **C.** NSG mice with orthotopic H2444 xenografts were administered with 14.8 MBq (400 μCi) of [^111^In]PD-L1-mAb and SPECT/CT images were acquired. Transaxial SPECT/CT images showing specific accumulation of activity in orthotopic H2444 xenograft at 72 h and 120 h after injection of the [^111^In]PD-L1-mAb and the corresponding histology **D.** Optical images of subcutaneous H2444 or H1155 xenograft acquired in the 800nm NIR channel **E.** and *ex vivo* biodistribution analysis of fluorescence intensity in tissues (*n* = 3) **F.** SPECT images were decay corrected and adjusted to the same maximum value to show the clearance of the imaging agent. The significance of the value is indicated by asterisk (*) and the comparative reference is the tumor with low PD-L1 expression. Arrows and circles depict tumors. *p < 0.05. **p < 0.01.

SPECT/CT imaging of mice showed high accumulation of radioactivity in subcutaneous H2444 tumors by 120 h, compared to H1155 tumors (Figure 4A), and the %ID/g for H2444 and H1155 tumors at 144 h were 7.46±0.12 and 3.63±0.57, respectively (Figure 4B). The tumor-to-muscle and tumor-to-blood ratios for H2444 tumors were 8.9±0.8 and 0.9±0.2, respectively. IHC evaluation showed strong PD-L1 immunoreactivity in H2444 tumors but not in H1155 tumors (Figure 4C), validating [^111^In]PD-L1-mAb specificity. In mice with orthotopic H2444 lung tumors, there was specific radioactivity accumulation delineating the tumors from normal lungs by 72 h, and significant enhancement in contrast at 120h (Figure 4D).

Optical imaging of H2444 and H1155 tumor bearing mice injected with NIR-PD-L1-mAb showed specific accumulation of fluorescence intensity in H2444 tumors (Figure 4E). This was confirmed by *ex vivo* biodistribution analysis, which showed a nearly two-fold increase in signal intensity in the H2444 tumors compared to H1155 (Figure 4F and Supplementary Figure 4). High fluorescence intensity was also observed in liver and kidneys. Collectively, these studies establish the feasibility of imaging endogenous PD-L1 expression in NSCLC tumors.

## DISCUSSION

Preclinical evaluation of a humanized radiolabeled anti-PD-L1 antibody, [^111^In]PD-L1-mAb, shows specific and increased uptake of radioligand in CHO tumors with stable PD-L1 expression compared to control CHO tumors. The *in vivo* specificity of [^111^In]PD-L1-mAb was confirmed by differential uptake in human breast and lung tumor xenografts with endogenous high and low PD-L1 expression, and by studies with the fluorophore-conjugated antibody, NIR-PD-L1-mAb. The results demonstrate a new and non-invasive means to detect PD-L1 expression in tumors with an antibody ready for clinical translation.

We first characterized the distribution of [^111^In]PD-L1-mAb and protein dose effect on antibody biodistribution in CHO tumors with constitutive PD-L1 expression [19]. Imaging and biodistribution studies showed that [^111^In]PD-L1-mAb uptake in the tumors was PD-L1 specific and that overall tissue distribution and tumor uptake were concentration dependent. Co-injection of unlabeled antibody prolonged circulation of [^111^In]PD-L1-mAb, significantly increased the tumor uptake and reduced spleen uptake. These studies suggest that pre-dosing with unlabeled antibody will improve tumor uptake of [^111^In]PD-L1-mAb and confirm reports from other PD-L1 antibodies [19, 20]. This could be possible because high doses of PD-L1 antibody could saturate the PD-L1 expression in splenocytes, increasing availability of the radioactive antibody to bind target sites within tumor. The high tumor-to-muscle we observed at 72 and 120 h, suggest that this would be the optimal time to image tumors with the [^111^In]PD-L1-mAb.

We then tested the specificity of the [^111^In]PD-L1-mAb in TNBC xenografts with endogenous PD-L1 expression. TNBC has one of the poorest survival rates [21]. In patients with metastatic TNBC, MPDL3280A has shown promising clinical activity, providing a new therapeutic opportunity for a cancer subtype that heavily relies on chemotherapy [2]. Better understanding of the TNBC response to immunotherapy could be improved by PD-L1 imaging. Our studies with [^111^In]PD-L1-mAb demonstrated specific uptake in MDAMB231 tumors with high PD-L1 expression, compared to that in SUM149 tumors that have low PD-L1 expression, suggesting that non-invasive PD-L1 detection is a viable option for TNBC. Similar results were also observed in other studies [19]. The values we report for tumor %ID/g in the MDAMB231 tumors are lower than those recently reported by Heskamp et al. (2015) [19]. In addition, our flow cytometry analysis showed that approximately 30% of MDAMB231 cells were positive for PD-L1 expression, which was markedly lower than the > 90% of positive cells reported by Heskamp et al. [19], accounting for the differences in %ID/g. Nevertheless, enhanced signal intensity accumulation and delineation of PD-L1 positive breast tumors is evident in our studies.

Immune checkpoint-targeted therapies have resulted in prolonged tumor regression and improved overall response rates, not only in immunogenic cancers such as melanoma and RCC, but also in cancers not believed to be immunogenic such as lung cancers. Treatment with MPDL3280A results in durable responses in NSCLC patients [6], and non-invasive PD-L1 detection in NSCLC may improve patient stratification. In an orthotopic lung tumor model (H2444), mice injected with [^111^In]PD-L1-mAb demonstrated SPECT signal in tumor regions that were spatially discrete from the normal lung, and aligned with tumor masses seen on CT by 72 h. Similarly, subcutaneous NSCLC xenografts showed specific uptake in H2444 tumors compared to H1155 tumors. The %ID/g values for H1155 were similar to CHO and SUM149 xenografts with low PD-L1 expression levels. However, %ID/g value for the H2444 tumor was found to be lower than what was anticipated based on the MFI values. Several factors can influence antibody uptake [22] and need to be investigated, including rapid tumor growth, enhanced permeability and retention, and tumor vascularity. Poor antibody delivery to the tumors could reduce therapeutic efficacy, despite their positive PD-L1 status. Non-invasive imaging, as we have demonstrated, could identify the disagreement between PD-L1 status and antibody delivery to the tumor. The clinical utility of such antibody imaging agents would be to use the radiolabeled antibody accumulation in the tumors to guide therapeutic antibody dosing and correlate that uptake with tumor response. This could be used to establish a relationship between tumor PD-L1 status and therapeutic response, which may have prognostic implications.

NIR intra-operative optical imaging has been used for staging and detection of tumor margins during cytoreductive surgery in ovarian cancer [23]. It is also becoming increasingly used to enhance tumor resection in brain surgery (using 5-aminolevulinic acid, scorpion venom, etc.) [24, 25], and new agents are emerging specific to a variety of other cancers, including prostate and breast [26-28]. With the NIR-PD-L1-mAb, PD-L1 positive tumor contrast was high in PD-L1 positive CHO-PDL1, MDAMB231 and H2444 tumors compared to CHO, SUM149 and H1155 tumors. That specificity suggests that NIR-PD-L1-mAb could be used for noninvasive PD-L1 detection by optical imaging.

Molecular imaging based “optical biopsies” are used for identification of lung nodules in a shorter period of time than standard IHC, and resection of those nodules with high success rates [29]. Similarly, NIR dye labeled EGFR antibody Cetuximab was used to visualize EGFR expression in head and neck squamous cell carcinomas [30]. NSCLCs show significant response rates for immune checkpoint targeted therapies and could benefit from availability of such technologies. Bronchoscopic imaging is routinely used for lung tumor diagnosis and staging but has not been used for PD-L1 expression status in lung tumors [31]. The specificity observed in the lung tumor models we tested indicates that NIR-PD-L1-mAb has the potential for bronchoscopic or thorascopic optical imaging of PD-L1 expression in lung tumors.

Imaging PD-L1 expression has recently been demonstrated using a mouse anti-human PD-L1 and hamster anti-mouse antibodies, and an engineered PD-1 derived fragment [19, 20, 32]. While those studies show the feasibility, the species from which the antibodies were derived and the near certainty of immunogenicity may curtail clinical translation, an experience observed with ProstaScint [33]. We chose MPDL3280A because it is in clinical trials and shows cross-reactivity to both human and mouse PD-L1 [13, 18]. Similarly cross-reactive Cetuximab was used to image tumor EGFR expression in patients [34]. Demonstrating the power of cross-reactive antibodies to evaluate tumor biology, Cetuximab was also used to detect colon adenocarcinoma in the setting of colitis in immunocompetent mouse models [35]. Such cross-reactivity is more desirable in the case of PD-L1 imaging agents because of the need for immunocompetent model systems to evaluate the immune responses. Importantly, results from preclinical studies using those cross-reactive antibodies, such as the one we have demonstrated using MPDL3280A, may guide clinical studies.

A variety of normal tissues express PD-L1 transcripts, including placenta, lung, liver, spleen, lymph nodes and thymus [7]. [^111^In]PD-L1-mAb cross-reactivity is reflected in the high radioactivity uptake observed in mouse spleen, lungs and liver in our present study. This is similar to the observations made by Josefsson et al. using an anti-mouse PD-L1 antibody [20]. Antibody cross-reactivity may also explain the significant differences in biodistribution reported in our study and the previous study using a mouse anti-human antibody by Heskamp et al. (2015) [19]. The consistently high radioactivity uptake observed in brown fat in our study, known to have immune cells, needs to be further investigated [36]. Because we used immunocompromised mouse models, the radioactivity uptake observed in immune related tissues could be less than one may see in immunocompetent mice. Nevertheless, our results provide an assessment of PD-L1 mAb distribution that could not be observed using either human-only or mouse-only reactive PD-L1 antibodies. Our data show human PD-L1 specific uptake of the antibody in the tumors, and mouse PD-L1 specific uptake in other tissues, and this provides a more detailed perspective on imaging PD-L1.

In summary, we have developed and evaluated nuclear and optical imaging agents for PD-L1 based on a humanized antibody. These represent two new tools that are ready for translation to patients undergoing immune checkpoint therapy. We have demonstrated PD-L1 specific accumulation of nuclear and fluorescent imaging agents in tumors with constitutive PD-L1 expression, and in TNBC and NSCLC xenografts with graded endogenous PD-L1 expression. The results demonstrate the feasibility of non-invasive PD-L1 imaging *in vivo*. PD-L1 has become an important target for cancer immunotherapy, and PD-L1-targeted antibodies have proved effective [6, 13]. Based on a humanized antibody that has shown therapeutic promise in patients, the presented data holds considerable potential for clinical translation. The patient management in advanced melanoma, breast and bladder cancers, in which PD-L1 antibodies have shown therapeutic efficacy but lack better response prediction and monitoring strategies, may particularly benefit from non-invasive PD-L1 detection at all the tumor sites.

## MATERIALS AND METHODS

### Reagents

The published sequence for anti-hPD-L1 monoclonal antibody (MPDL3280A, clone YW243.55.S70) was used to guide the synthesis, performed by Evitria (Zurich, Switzerland) [13, 18]. All chemicals were purchased from Sigma-Aldrich or Fisher Scientific unless otherwise specified. The IRDye^®^ 800CW NHS ester, CHX-A”- DTPA and [^111^In]Cl_3_ were purchased from LI-COR (catalog # 929-70020; Lincoln, Nebraska), Macrocyclics Inc. (catalog # B355; Dallas, TX, USA,) and Nordion (Vancouver, BC, Canada), respectively. All cell culture related reagents were purchased from Invitrogen, unless otherwise specified.

### Cell lines

Four cell lines NCI-H2444 (NSCLC, PD-L1^high^), NCI-H1155 (NSCLC, PD-L1^low^), MDAMB231 (TNBC, PD-L1^high^) and CHO-K1, (henceforth referred to as H2444, H1155, MDAMB231 and CHO respectively), were purchased from the American Type Culture Collection (ATCC) and passaged for fewer than 3 months after which new cultures were initiated from vials of frozen cells. The SUM149 (TNBC, PD-L1^low^) cell line was kindly provided by Dr. Stephen P. Ethier, Medical University of South Carolina, and authenticated by STR profiling at the Johns Hopkins genetic resources facility. SUM149 cells were maintained in Ham's F-12 medium with 5% FBS, 1% P/S and 5 μg/mL insulin, and 0.5 μg/mL hydrocortisone. All other cell lines were cultured in ATCC recommended media in an incubator at 37°C in an atmosphere containing 5% CO_2_. The CHO-PDL1 cell line generation and maintenance are described in Supplementary Methods.

### Flow cytometry

Cells in suspension were harvested by centrifugation with adherent cells detached using enzyme-free, PBS-based cell dissociation buffer (Gibco). The harvested cells were washed twice with flow cytometry buffer (1xPBS with 2 mM EDTA and 0.5% FBS). Cells were stained with anti-human PD-L1 antibody conjugated with phycoerythrin (PE) (catalog #557924, Becton Dickinson) according to the manufacturer's protocol and were analyzed on a FACSCalibur flow cytometer (Becton Dickinson). At least 20,000 events were recorded and analyzed using FlowJo software (Tree Star).

### Animal models

Animal studies were performed according to the protocols approved by the JHU Animal Care and Use Committee (ACUC). Six-to-eight weeks old, female, non-obese diabetic severe-combined immunodeficient gamma (NSG) mice were obtained from the JHU Immune Compromised Animal Core. Mice were implanted subcutaneously in the upper flanks with CHO-PDL1 (10×10^6^), CHO (10×10^6^), H2444 (10×10^6^), or H1155 (2×10^6^) cells and orthotopically in the upper mammary fat pads with MDAMB231 (2×10^6^), and SUM149 (2×10^6^) cells in 100 μL of HBSS containing 50% matrigel (Corning). Mice were used for imaging or biodistribution experiments when the tumors reached a volume of 200-300 mm^3^.

Orthotopic mouse models of lung tumors were generated by injecting lung cancer cells into the left lung of NSG mice. A 1 cm skin incision was made on the left scapula of the anesthetized mouse and the thoracic muscles were separated to expose the costal layer. The H2444 cells, (1×10^6^ in 30 μL HBSS containing 50% matrigel), were injected directly though the intercostal space into the left lung, by using a 29G needle on a 0.5 mL syringe. Skin incisions were closed by sutures and antibiotics were applied topically. Growth of the orthotopic lung tumor was monitored by CT imaging.

### Preparation of radiolabeled antibody

The PD-L1 mAb was reacted with N-[(R)-2-Amino-3-(p-isothiocyanato-phenyl) propyl]-trans-(S,S)-cyclohexane-1,2-diamine-N,N,N',N”,N”-pentaacetic acid (CHX-A”-DTPA, #B355, Macrocyclics, Inc.) in 0.1 M sodium bicarbonate buffer (pH = 9.5) at 1:50 mAb:CHX-A”-DTPA molar ratio, for 1 h at room temperature. Unconjugated CHX-A”-DTPA was removed using a Zeba™ spin desalting column, pre-equilibrated with 0.1 M 2-(N-morpholino)ethanesulfonic acid (MES) buffer. The DTPA-conjugated mAb was further purified on an Amicon ultra-4 centrifugal filter with 10k Da MWCO (# UFC801024, EMD Millipore, Billerica), using 0.1 M ammonium acetate (pH 5.5). Conjugation of CHX-A”-DTPA to antibody was confirmed by matrix assisted laser desorption ionization mass spectrometry. Radiolabeling of mAb-DTPA with ^111^In was carried out in 0.3 M ammonium acetate (pH 5.5) for 1 h at 37°C, under metal free conditions. The resulting [^111^In]PD-L1-mAb was incubated with ethylenediaminetetraacetic acid (EDTA) at a final concentration of 5 mM for 5 min to chelate unbound ^111^In^3+^, and further purified on a desalting column. Radiochemical purity was tested by instant thin-layer chromatography (ITLC) (catalog # 61885, Pall Life Science) using citrate-phosphate-dextrose solution as a mobile phase (Supplementary Figure 1A-1C).

### Preparation of NIR dye conjugated antibody

Synthesis of PD-L1 mAb conjugated with Licor800 dye (NIR-PD-L1-mAb) was performed according to manufacturer's protocol. Briefly, 3 mg of mAb dissolved in 400 μL of 1xPBS were mixed with 4 μL of DMSO containing 0.09 mg of IRDye^®^ 800CW NHS ester. The reaction mixture was gently stirred for 2 h at room temperature, followed by purification using PBS pre-equilibrated Zeba™ spin desalting columns, 7K MWCO, 0.5 mL (#89882, Thermo Fisher Scientific). Analysis of the NIR-PD-L1-mAb was carried out using a Nanodrop 2000 UV-vis spectrophotometer (Thermo Fisher Scientific), which indicated 1:2 mAb:IR800 molar ratio (Supplementary Figure 1D).

### *In vitro* binding assay and immunoreactive fraction (IF) determination

*In vitro* binding of [^111^In]PD-L1-mAb to CHO-PDL1, CHO, MDAMB231, SUM149, H2444, and H1155 cells was determined by incubating 1 μCi of [^111^In]PD-L1-mAb with 1×10^6^cells (in triplicate for each cell line) for 1h at 37°C. PD-L1 blocking was performed by adding a 10-fold molar equivalent excess of the non-labeled mAb. After incubation, cells were washed three times with cold PBS prior to counting on an automated gamma counter (1282 Compugamma CS, Pharmacia/LKBNuclear, Inc., Gaithersburg, MD).

To determine the immunoreactive fraction (IF), binding of [^111^In]PD-L1-mAb to 4, 2, 1, 0.5 and 0.25 million CHO-PDL1 cells was carried out as described above. IF was calculated using the Lindmo assay [37]. All *in vitro* studies were performed in triplicate and repeated three times.

### SPECT/CT imaging and analysis

Whole-body SPECT-CT images were acquired on an X-SPECT small animal SPECT/CT system (Gamma Medica Ideas, Northridge, CA, USA) as described previously [38]. Briefly, after an intravenous injection of approximately 400 μCi of [^111^In]PD-L1-mAb (100 μg of antibody, *n* = 3), images were acquired at the specified time points in mice. The tomographic data were acquired in 64 projections over 360^o^, at 45 s per projection, using medium energy pinhole collimators. CT images were acquired in 512 projections to allow anatomic co-registration. Images were reconstructed using the ordered subsets-expectation maximization algorithm, and 3D volume rendered decay corrected images were generated using Amira 5.5.0 software (Visage Imaging Inc.).

### *Ex vivo* biodistribution

Specific activity of [^111^In]PD-L1-mAb was optimized for *in vivo* distribution and tumor uptake in mice harboring CHO-PDL1 and CHO xenografts. Mice (*n* = 3-5/group) were injected intravenously with 40 μCi of [^111^In]PD-L1-mAb alone (approximately ∼ 8.5 μg of the protein), or in combination with 10, 30, and 90 μg of unmodified PD-L1-mAb. For the blocking study, mice were pre-injected with 1.5 mg of unmodified mAb 30 min before injection of [^111^In]PD-L1-mAb. Blood, tumors, and selected tissues were harvested, weighed and counted in an automated gamma counter (1282 Compugamma CS, Pharmacia/LKBNuclear, Inc.) at 48 h after the [^111^In]PD-L1-mAb injection. Following this optimization, all other biodistribution studies were performed with 40μCi [^111^In]PD-L1 mAb combined with 30 μg of unmodified mAb.

Biodistribution studies were also carried out to confirm the imaging study results in CHO/CHO-PDL1, SUM149/MDAMB231 and H1155/H2444 xenograft models with low or high expression of PD-L1 respectively, at various time points after [^111^In]PD-L1-mAb injection. The percentage of injected dose per gram of tissue (%ID/g) values were calculated based on signal decay correction and normalization to external ^111^In standards, which were measured in triplicate. Biodistribution data shown is mean ± the standard error of the mean (SEM).

### Optical imaging

PD-L1 expression in different tumor models was assessed by optical imaging using NIR-PD-L1. The NIR-PD-L1 (22 μg) was injected into the tail vein of the mice (*n* = 3-5) bearing tumors with low and high expression of PD-L1. Mice were anaesthetized with isoflurane and serial images of the dorsal, left lateral, ventral and right lateral surfaces were captured using the Pearl Impulse Imager in white light and 800 nm channels (Software v2.0, LI-COR Biosciences) at 24, 48, 72, 96 and 120 h post injection. On day 5 after the injection of NIR-PD-L1-mAb, mice were euthanized and tumors and selected tissues were dissected and imaged *ex vivo*. To quantify the signal, equal sized regions of interest (ROIs) were drawn on tumors and tissues and on an area outside the mouse and representative of background. Mean signal intensity in each ROI was normalized by subtracting the background signal, and used for statistical analysis. Data shown is mean fluorescence intensity values ± SEM.

### Immunohistochemistry

Tumor sections were evaluated for PD-L1 expression by immunohistochemistry (IHC). Harvested tumors were fixed in 10% neutral buffered formalin, embedded in paraffin, and 4 μm thick sections were obtained on slides. After deparaffinizing with xylene and alcohol gradients, antigen retrieval was done using 10 mM citrate buffer, pH 6.0 (#S1699, Dako target retrieval solution). Tumor sections were then treated with 3% H_2_O_2_ for 10 minutes, blocked with 5% goat serum for 1 h, and then incubated with a primary anti-human PD-L1 antibody (#13684, Cell Signaling) at 1:500 dilution at 4°C overnight. Subsequently, using Dako CSAII Biotin-free Tyramide Signal Amplification System kit, slides were incubated with secondary antibody, amplification reagent, and with anti-fluorecein-HRP. Finally, staining was carried out by adding DAB chromogen. Sections were counterstained with hematoxylin, followed by dehydration with alcohol gradients, xylene washes and mounted with a cover slip.

### Data analysis

Statistical analysis of *in vitro* receptor binding assay data and *ex vivo* biodistribution data were performed with Graphpad Prism 6 software using an unpaired two-tailed t test. When *P* < 0.05, the difference between the compared groups was considered to be statistically significant.

## SUPPLEMENTARY MATERIAL FIGURES


