# Supplementary material for: A humanized antibody for imaging immune checkpoint ligand PD-L1 expression in tumors
Source: Oncotarget. 2016 Feb 1;7(9):10215–27. doi: 10.18632/oncotarget.7143 (PMC4891115; doi:10.18632/oncotarget.7143)
Supplement: Supplementary file 1 [file oncotarget-07-10215-s001.pdf]

A humanized antibody for imaging immune checkpoint ligand PD-L1 expression in tumors

Supplementary Material

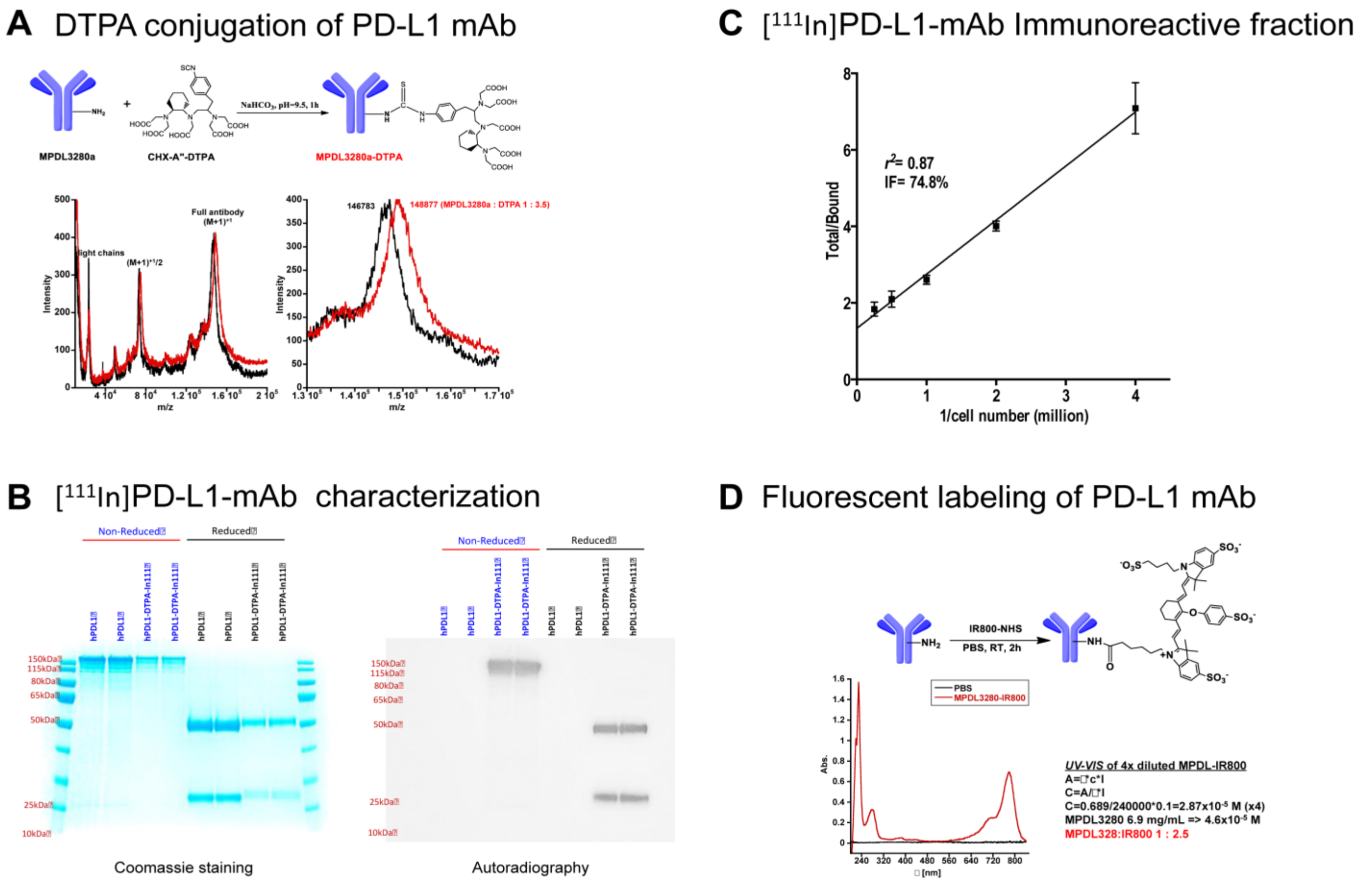

Supplementary Figure 1: **Characterization of [^{111}In]PD-L1-mAb and NIR-PD-L1-mAb.** MALDI-TOF spectra of unmodified PD-L1-mAb and DTPA conjugated PD-L1-mAb (**A**). SDS-PAGE analysis and autoradiography of [^{111}In]PD-L1-mAb confirmed antibody integrity and radiolabeling **(B)**. IF determination by Lindmo assay **(C).** UV/Vis spectrum of NIR-PD-L1-mAb **(D).**

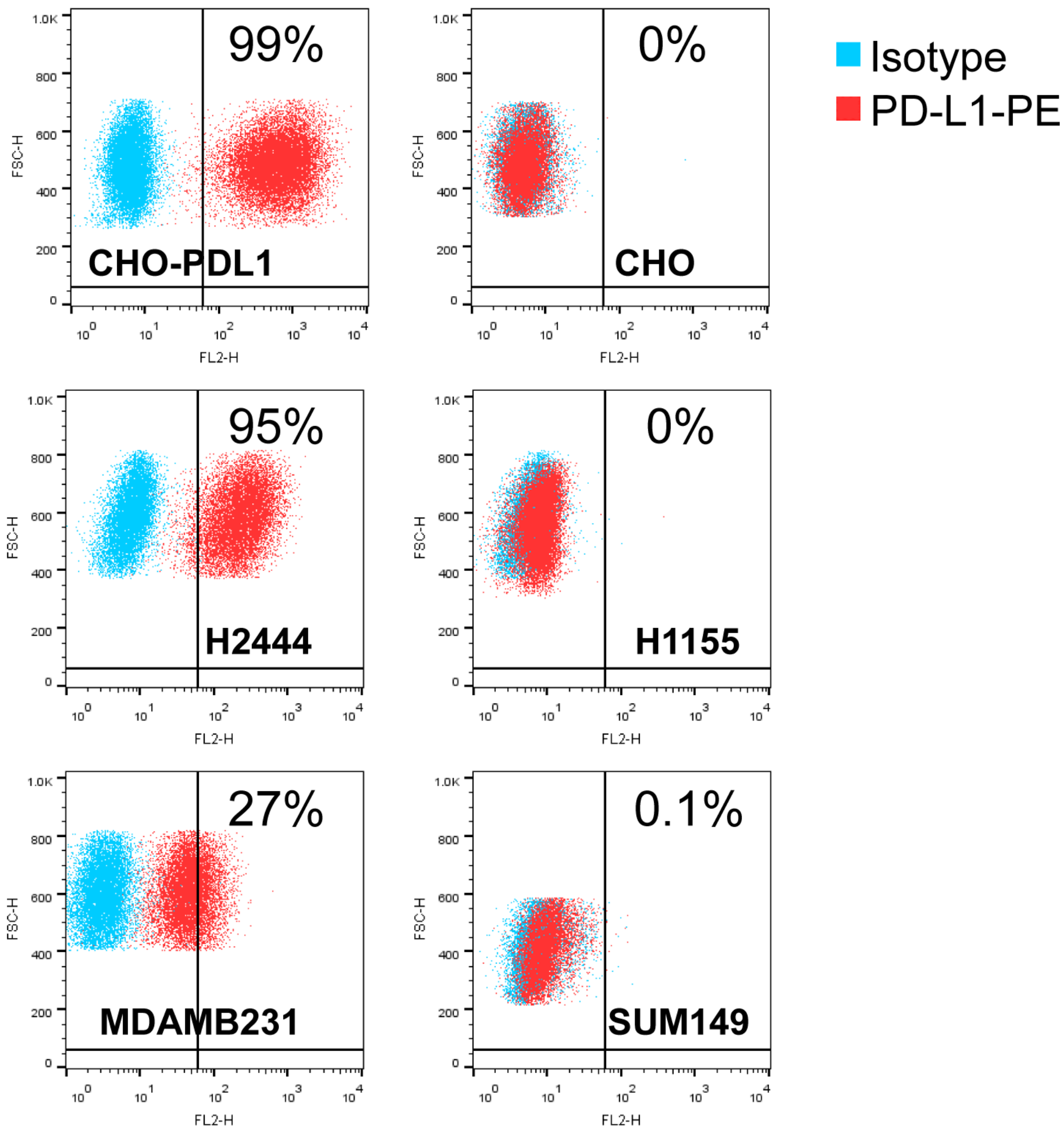

Supplementary Figure 2: PD-L1 expression in cell lines as determined by flow cytometry.

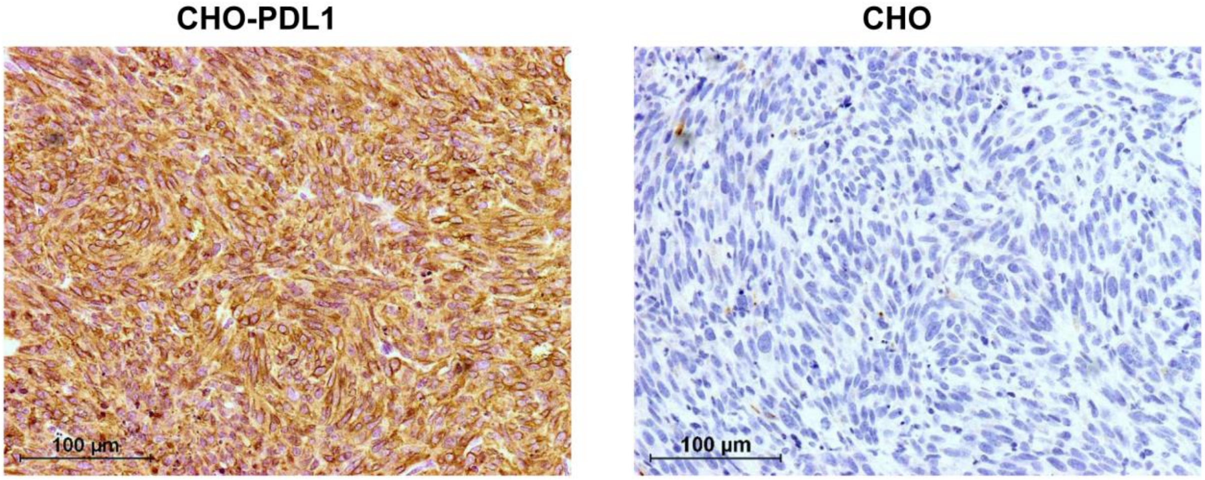

Supplementary Figure 3: Immunohistochemical analysis of PD-L1 expression in CHO-PDL1 and CHO tumors.

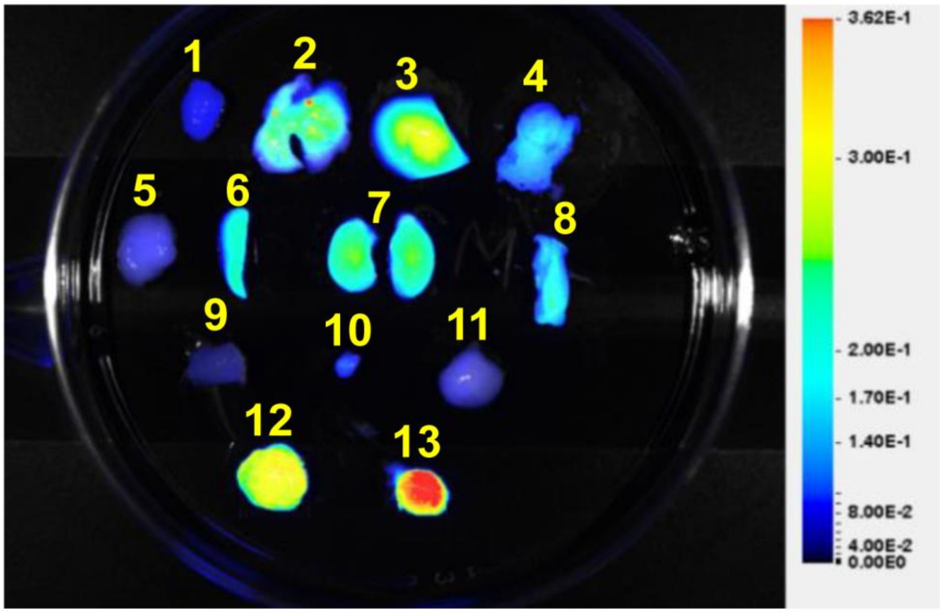

1. Heart
2. Lungs
3. Liver
4. Stomach
5. Pancreas
6. Spleen
7. Kidneys
8. Small Intestine
9. Muscle
10. Bladder
11. Fat
12. H1155
13. H2444

Supplementary Figure 4: *Ex vivo* biodistribution analysis of the NIR-PD-L1-mAb at 120 h after injection in H2444 and H1155 subcutaneous tumor models.

Supplementary Methods:

Generation of PD-L1 stable transfected CHO cell line

TrueClone plasmid containing human PD-L1 cDNA ORF was purchased from Origene (#SC115168). For transfection, CHO cells were grown to 60-80% confluence and growth medium was replaced with antibiotic-free growth medium. Plasmid DNA and Lipofectamin2000 (Invitrogen) were diluted in Opti-MEM I reduced serum medium (Gibco), mixed gently and incubated for 25 minutes at room temperature to form DNA-Lipofectamine LTX complexes. This mixture with complexes was added directly to the CHO cells and the cells were kept at 37°C in a CO_2 incubator. After 24 h, medium was replaced with fresh growth medium containing 2 mg/mL G418 (Corning) as selection antibiotic. Transfected cells were harvested, fluorescently labeled with anti-human PD-L1 antibody conjugated with phycoerythrin (PE) and PD-L1 positive cells were sorted on a FACSAria flow cytometer (Becton Dickinson). Sorted cells were plated to derive clones of single cell origin. PD-L1 expression in different clones were analyzed, and the positive clone with highest PD-L1 expression was selected for further studies. This positive clone (CHO-PD-L1) was maintained in F-12K medium with 10% FBS, 1% P/S and 2 mg/mL G418.

SDS-PAGE Analysis and Autoradiography

Unmodified or radiolabeled antibodies were electrophoresed in 1mm NuPAGE Novex Gel, either in reducing condition (sample with 0.7M 2-mercaptoethanol, heated at 95°C for 2 min before loading) or in native non-reducing condition (sample without 2-mercaptoethanol and no-heating). After completion of the electrophoresis, the gel was stained with colloidal Coomassie G-250 as per manufacturer's protocol (SimplyBlue™ - Life Technologies). The stained gel was then exposed to X-ray film overnight for autoradiography.

Matrix-Assisted Laser Desorption Ionization-Time-of-Flight (MALDI-TOF)

Spectra of unmodified antibody and conjugated with DTPA were recorded on a Voyager DE-STR spectrophotometer, using 2,5- dihydroxybenzoic acid (DHB) as a matrix. First 10 µL of matrix at concentration of 20 mg/mL was mixed with 10 µL of antibody at concentration of 2 mg/mL. Then 1 µL of resulting mixture was placed on the target plate (in triplicate) and evaporated. Matrix was dissolved in 50% MeOH and 0.1% TFA aqueous solution and protein in DI water. Number of shots and laser power was adjusted according to spectrum quality.
